# Evaluation of Potentially Toxic Metal Contamination of Local Medicinal Plants and Extracts Sold in Ibadan, Nigeria

**DOI:** 10.5696/2156-9614-7.14.23

**Published:** 2017-06-22

**Authors:** Gilbert U. Adie, Adedoyin Adekunle

**Affiliations:** Department of Chemistry, Faculty of Science, University of Ibadan, Ibadan, Nigeria

**Keywords:** medicinal plants, heavy metals

## Abstract

**Background.:**

Extracts from medicinal plants have been widely used in the treatment of public health ailments, however, medicinal plants may be grown in polluted soil/water environments. Many of these plants are harvested and processed by local and illiterate natural healers and other vendors in an unhygienic manner. This results in the possibility of contamination with potentially toxic metals from the environment.

**Objectives.:**

This study evaluated the concentrations of lead (Pb), cadmium (Cd), chromium (Cr), nickel (Ni), copper (Cu) and zinc (Zn) in 25 samples of different medicinal plants procured from stores in open markets in Ibadan, Nigeria.

**Methods.:**

After procurement, the samples were air dried, pulverized and dry ashed. All ashes were dissolved with dilute acid solutions, filtered and the filtrates were stored for metal analysis. Samples with elevated metal concentrations were extracted with water and alcohol solutions to mimic the medicinal extracts obtained from these plants. Metal concentrations were analysed in all extracts and filtrates using atomic absorption spectrophotometry.

**Results.:**

Metal concentrations (mg/kg dry weight) in all samples ranged as follows: Cu, 0.04 - 9.44; Zn, 0.36 – 35.4 and Pb, below detection limits (BDL) – 6.15. The concentrations of Cd, Cr and Ni in all samples were BDL. The concentrations of all metals in the samples were within accepted limits set for medicinal plants according to international regulatory bodies. All ethanol extracts and 1 out of 4 water extracts contained Pb.

**Conclusions.:**

Medicinal plants could pose chronic metal toxicity effects from continual bioaccumulation along the food chain. Furthermore, extraction of active ingredients with water was deemed to be safer than the use of alcohol extracts. Continuous monitoring of these medicinal plant materials is needed.

## Introduction

Medicinal plants are plants that contain in one or more of their parts substances that can be used for therapeutic purposes.^1^,^2^ According to the World Health Organization, over 70–80% of the world's population living in rural areas relies on non-conventional medicine for the treatment of their ailments.^3^ Many of these individuals believe that medicinal plants are natural and thus safer than allopathic drugs.^4^ Furthermore, there has been a two-fold increase in the demand for medicinal plants on the international market. The World Health Organization (WHO) estimated that within the last 15 years, the demand for medicinal plants has reached around $14 billion annually and could reach $5 trillion by 2050.^5^

However, owing to the nature and sources of these medicinal plants, they are sometimes contaminated with toxic metals such as lead, arsenic, mercury and cadmium, which pose serious health risks to consumers. It is critical to examine the source materials and extracts for toxic metals in order to ensure the safety, efficacy and quality of medicinal plants.

There have been many studies screening a wide range of medicinal plants across the globe for potentially toxic metal concentrations. Mekassa and Chandravanshi (2015) studied the levels of selected essential and non-essential metals in seeds of korarima (Aframomum corrorima) cultivated in Ethiopia.^6^ Evaluation of heavy metal levels in spices and herbs available on the Polish market was carried out by Krejpcio et al. (2007).^7^ Cadmium and micronutrient levels in spices commonly consumed in Turkey have been monitored by Ozkutlu et al. (2006).^8^ A study on microbial and heavy metals contamination of medicinal medicines has also been reported.^9^ Many other studies have estimated the concentrations of potentially toxic metals in plants with medicinal value.^10–21^

Medicinal plants can accumulate heavy metals when grown on polluted environmental media such as roadsides and metal mining and smelting operations.^22^ Furthermore, heavy metal contamination in medicinal herbs can also be due to anthropogenic processes involving application of synthetic fertilizers, organic manures, lime and industrial effluents that contaminate the agro ecosystem or during transportation and unhygienic storage conditions.^12^,^16^ The use of fertilizers containing cadmium (Cd), organic mercury or lead-based pesticides could also contaminate medicinal plant materials with toxic metals.^23^ Gogtay et al., 2002 reported that potentially toxic metals are sometimes deliberately added to ‘Ayurvedic’ and traditional Chinese medicines because they are thought to have therapeutic properties.^24^

In Nigeria, as in other parts of the world, local medicinal plants/extracts could be contaminated by potentially toxic metals either by uptake from contaminated environmental media or during handling, processing, and storage. Furthermore, in Nigeria, a greater percentage of local medicinal plants are marketed by untrained and illiterate natural healers and other vendors in an unhygienic manner. There is little or no information on the concentrations of toxic metal concentrations like lead (Pb), mercury (Hg), arsenic (As), Cd, chromium (Cr), nickel (Ni), copper (Cu), and zinc (Zn) in indigenous medicinal plants/extracts. This study was designed to determine the concentrations of Cu, Zn, Pb, Cd, Cr and Ni in 25 samples of frequently used medicinal plants obtained from local vendors in Ibadan, Nigeria. The potentially toxic metals determined in this study were chosen because the method of their analysis was readily available. However, important metals like Hg and As were not determined because cold vapor and hydride generation techniques that usually atomize these metals were not available. The metal concentrations obtained from this study could form part of the baseline data for the metals in Nigeria.

## Methods

### Sample Collection and Identification

Twenty-five different medicinal plant samples were purchased from the stores of local vendors in the popular Oje Market in Ibadan. The plants were already in the shops/stores of the vendors at the time of purchase. The vendors normally obtain these plants from bushes throughout the year when they are available, dry them and store them for sale or treatment of ailments. The purpose of this study was to buy the plants from the local general markets to determine the toxicity level of the analyzed metals to which the public is exposed when they consume extracts made from these plants. The plants were selected after interviews with local vendors determined them to be the most commonly used. Table 1 indicates the various therapeutic uses of these plants. The plants were identified at the Department of Botany, University of Ibadan.

**Table 1:**
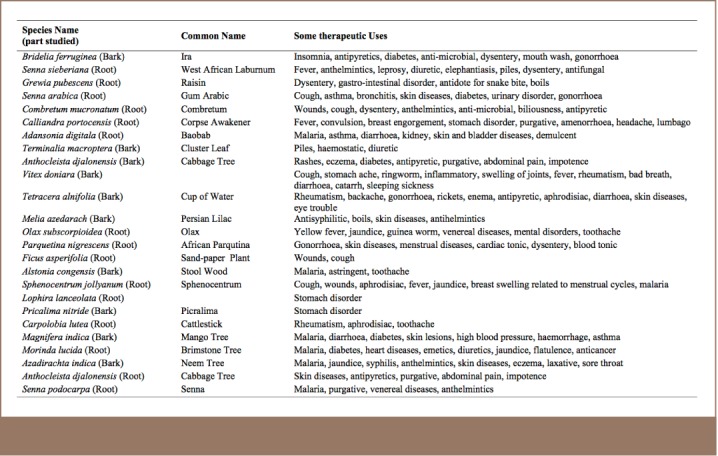
Medicinal Plants and Their Associated Therapeutic Uses

**Abbreviations**
*As*: Arsenic
*BDL*: Below detection limit
*Cd*: Cadmium
*Cr*: Chromium
*Cu*: Copper
*Hg*: Mercury
*Ni*: Nickel
*Pb*: Lead
*Zn*: Zinc

### Preparation and Digestion of Samples

The plant materials were air-dried at room temperature in the laboratory for one week. After drying, they were chopped into smaller pieces with the aid of a stainless steel cutlass and then pulverized by an electric grinder fitted with a stainless steel blade. The samples were sieved using a 2 mm mesh size sieve. Equipment was cleaned in between preparing each sample. Exactly 5 g each of the previously sieved samples were muffled in a furnace with a gradual rise in temperature until the temperature was stabilized at 550° C for 4—6 hours to ensure that all organic components of each sample were completely oxidized as demonstrated by a uniform ash for each sample. The ash arising from each sample was dissolved with nitric acid solution and subsequently filtered with Whatman No. 1 filter paper directly into a 25 mL standard flask and made up to mark with distilled water. A set of blank samples were also carried through the process to check for impurities from the reagents and/or environment. The solutions for all samples after filtration and making up to volume were stored in previously acid washed plastic containers and refrigerated at 4° C until analysis by atomic absorption spectrophotometry (AAS).

### Extraction Studies

Samples that showed appreciable concentrations of Pb after AAS analysis were further subjected to extraction with water and alcohol to check the amount of Pb that could be extracted, as these medicinal plants are normally consumed as extracts obtained by soaking in water and/or alcohol. Four plants: Bridelia ferruginea (2.30 mg/kg), Andasonia digitala (1.60 mg/kg), Sphenocentrum jollyanum (6.15 mg/kg) and Morinda lucida (4.85 mg/kg) were found to contain appreciable concentrations of Pb. Exactly 53 g each of the four plants were separately soaked in plastic containers with distilled water and ethanol solution in such a manner that all the plant materials were completely submerged in the solutions. Extracts were withdrawn from the solutions after 24 hours, 48 hours, 72 hours and 96 hours. They were concentrated on a water bath to less than 25 mL and filtered using Whatman No.1 filter papers into 25 mL standard flasks and made up to mark. These solutions were analysed for Pb using AAS. Blanks containing no sample were also carried through the process and analysed for Pb.

### Recovery Study

A recovery study was performed to validate the method of analysis. Normally in a recovery study, a few samples that have been previously analysed and have varying concentrations of the metals are chosen and taken through the recovery test to validate the method used. In this study, a recovery experiment was conducted on two plant species, Bridelia ferruginea and Andasonia digitala, which had been previously analyzed. The % recovery ranged from 94 to 105% for all metals. This was within the acceptable limit of 100±10%.^25^

## Results

### Metal Concentrations in Studied Medicinal Plants

The summary of metal concentrations in the analysed medicinal plants is presented in Table 2. The metal concentrations in order of decreasing magnitude were Cu > Zn > Pb > Cd = Cr = Ni. Out of the 25 medicinal plant samples, Cu and Zn were detected in all of the samples, Pb was detected in only 4 of the samples, and Cd, Cr and Ni were not detected in any of the samples.

**Table 2:**
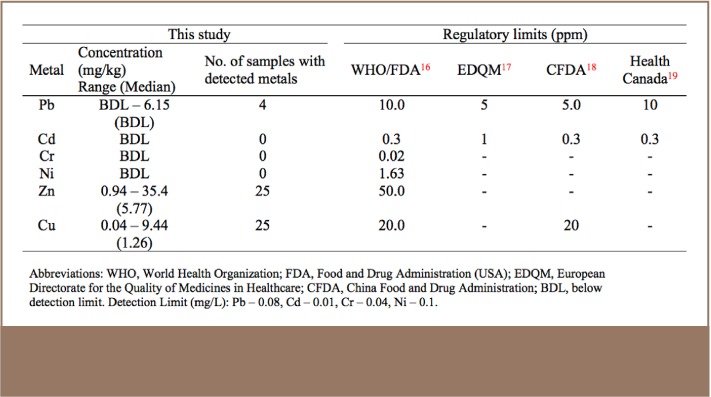
Metal Concentrations in Medicinal Plants Compared with Selected Regulatory Limits

### Comparison of Metal Concentrations

Table 3 compares metal concentrations found in this study with similar studies from the literature. A general analysis of the results in Table 3 indicates that Pb and Cd concentrations were elevated in most medicinal plants compared with permissible limits globally, but As and Hg were found to be within permissible limits.

**Table 3:**
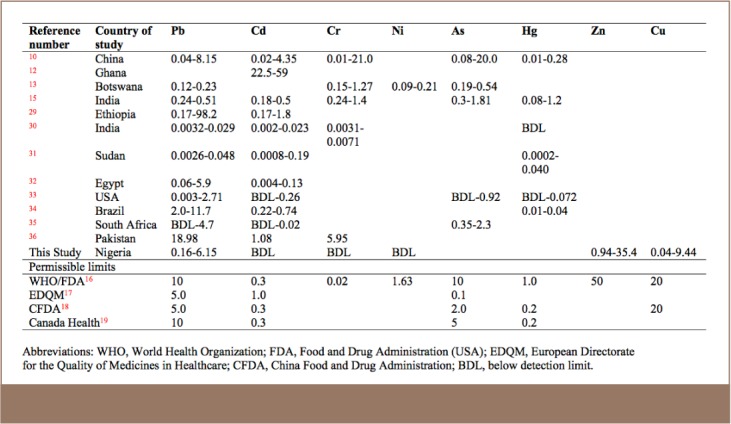
Metal Concentrations (mg/kg dry weight) Found in this Study Compared with Previous Studies

### Extraction of Pb From Medicinal Plant Samples Using Water and Ethanol

Table 4 presents Pb concentrations in extracts obtained from soaking samples that were found to contain Pb to determine the amount that could leach out. This was done to mimic the method in which local vendors extract the active ingredients from the bulk sample.

**Table 4:**
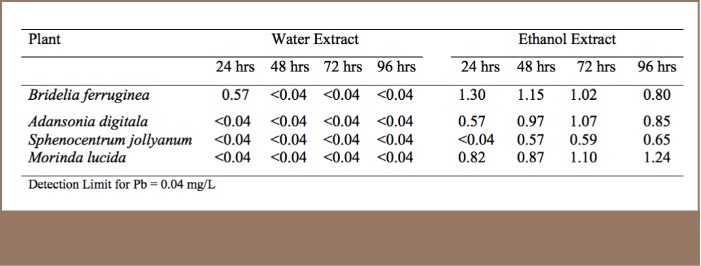
Concentration of Pb (mg/L) in Water and Ethanol Medicinal Plant Extracts

### Correlation Studies

Table 5 shows Spearman correlations at a 95% significant level between Pb, Cu and Zn to examine the relationship of their sources of origin. The p values for all pairs except Cu/Zn (p = 0.013) were > 0.05, implying no significant differences between the pairs. This could mean that Cu/Pb and Zn/Pb had related sources.

**Table 5:**
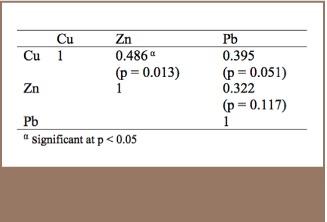
Spearman Correlations

## Discussion

The range and median of metal concentrations (mg/kg) in all plant samples were as follows: Zn: 0.94–35.4 (5.77), Cu: 0.04–9.44 (1.26), Pb: below detection limit (BDL)–6.15 (BDL). Cadmium, Cr and Ni were all below detection limits in all plant samples.

The occurrence of Zn and Cu in all study samples was not surprising because these metals are essential metals that are naturally present in plants and play a role in many metabolic and enzymatic processes in plants. Lead was detected in four of the plants sampled: Bridelia ferruginea (2.30 mg/kg), Andasonia digitala (1.60 mg/kg), Sphenocentrum jollyanum (6.15 mg/kg) and Morinda lucida (4.85 mg/kg). Lead is ubiquitous in the environment and lead contamination could have occurred during growing, harvesting, transporting, processing or storing. The concentrations of all metals compared with regulatory limits set by well known international agencies (*Table 2*) were within permissible limits. The presence of Pb in some plant samples calls for concern. Consumption of materials with even trace amounts of Pb and other potentially toxic metals should be regulated, as these metals tend to gradually bioaccumulate in human tissues.^37^

Some factors that could influence the metal concentrations across different studies include analytical methods and equipment used, region, type and nature of samples. The elevated concentrations of Pb and Cd may be due to industrial applications of these metals, including use in batteries, paints, household and electronic products. Wastes arising from these products in many parts of the world, especially Asia and Africa, are disposed of in environmentally unsound ways.^38^ Therefore, medicinal plants could be easily contaminated by these metals along the process chain ranging from planting, harvesting to storing and packaging for sale.

On the one hand, Pb was detected in water extracts in only one out of the four samples analyzed as shown in Table 4. This could indicate that minimal amounts of Pb and other metals are extracted by water. On the other hand, Pb was detected in three of the four samples extracted with ethanol. Furthermore, Pb was detected in alcohol extracts after soaking for 96 hours (4 days). The timing was selected to mimic the average time that local vendors of medicinal extracts soak plant materials before replacing them with fresh ones. The high extraction levels exhibited with the ethanol solution is an indication that some of the Pb was held within the organic matrices of the sample which could have dissociated by ethanol. Lead concentrations in ethanol extracts ranged from <0.04–1.24 mg/L. These levels were within acceptable limits, but continuous exposure to these small concentrations can bioaccumulate in human tissues and pose chronic toxic effects. Therefore, water extraction is recommended for medicinal plant extracts as this method appears to be safer compared to alcohol extraction.

## Conclusions

Twenty-five frequently used medicinal plant samples acquired from local vendors in Ibadan, Nigeria were analyzed for contamination by potentially toxic metals, namely Pb, Cd, Cr, Ni, Cu and Zn. All samples contained Cu and Zn; Pb was detected in 4 of the 25 samples; and Cd, Cr and Ni were not detected in any of the samples. The concentrations of the detected metals were within the ranges of similar studies as well as the regulatory limits set for medicinal plant materials. In addition, Pb was analyzed in water and ethanol extracts obtained from the 4 samples previously containing Pb. Ethanol solutions revealed a stronger extraction power compared with water, possibly due to the release of Pb bound to organic matrices that might have been dissociated by ethanol. In conclusion, extracts from the selected medicinal plants might not pose an immediate danger to consumers. Furthermore, water extracts appear to be safer than ethanol extracts. Finally, consumption of extracts from medicinal plants in Ibadan should be done with caution, as the small concentrations of these potentially toxic metals have the ability to bioaccumulate in human tissues to concentrations that could become lethal.
